# Trait-based approaches for understanding microbial biodiversity and ecosystem functioning

**DOI:** 10.3389/fmicb.2014.00251

**Published:** 2014-05-27

**Authors:** Sascha Krause, Xavier Le Roux, Pascal A. Niklaus, Peter M. Van Bodegom, Jay T. Lennon, Stefan Bertilsson, Hans-Peter Grossart, Laurent Philippot, Paul L. E. Bodelier

**Affiliations:** ^1^Department of Microbial Ecology, Netherlands Institute of Ecology (NIOO-KNAW)Wageningen, Netherlands; ^2^Department of Chemical Engineering, University of WashingtonSeattle, WA, USA; ^3^Ecologie Microbienne, CNRS, INRA, Université de Lyon, Université Lyon 1, UMR 5557, USC 1193Villeurbanne, France; ^4^Institute of Evolutionary Biology and Environmental Studies, University of ZurichZurich, Switzerland; ^5^Subdepartment of Systems Ecology, Department of Ecological Sciences, VU University AmsterdamAmsterdam, Netherlands; ^6^Department of Biology, Indiana UniversityBloomington, IN, USA; ^7^Limnology and Science for Life Laboratory, Department of Ecology and Genetics, Uppsala UniversityUppsala, Sweden; ^8^Leibniz-Institute for Freshwater Ecology and Inland FisheriesBerlin, Germany; ^9^Institute for Biochemistry and Biology, Potsdam UniversityPotsdam, Germany; ^10^INRA, UMR 1347 AgroecologieDijon, France

**Keywords:** functional traits, ecosystem function, ecological theory, study designs, microbial diversity

## Abstract

In ecology, biodiversity-ecosystem functioning (BEF) research has seen a shift in perspective from taxonomy to function in the last two decades, with successful application of trait-based approaches. This shift offers opportunities for a deeper mechanistic understanding of the role of biodiversity in maintaining multiple ecosystem processes and services. In this paper, we highlight studies that have focused on BEF of microbial communities with an emphasis on integrating trait-based approaches to microbial ecology. In doing so, we explore some of the inherent challenges and opportunities of understanding BEF using microbial systems. For example, microbial biologists characterize communities using gene phylogenies that are often unable to resolve functional traits. Additionally, experimental designs of existing microbial BEF studies are often inadequate to unravel BEF relationships. We argue that combining eco-physiological studies with contemporary molecular tools in a trait-based framework can reinforce our ability to link microbial diversity to ecosystem processes. We conclude that such trait-based approaches are a promising framework to increase the understanding of microbial BEF relationships and thus generating systematic principles in microbial ecology and more generally ecology.

## BEF research—a brief overview

The relationship between biodiversity and ecosystem functioning (BEF) (Table 1) is complex and understanding this elusive link is one of the most pressing scientific challenges with major societal implications (Cardinale et al., 2012). However, previous studies established controversial views on BEF relationships, using approaches which experimentally manipulated biodiversity on the one hand and comparative approaches that correlate diversity and ecosystem functioning across treatments or natural gradients on the other hand (Hooper et al., 2005; Balvanera et al., 2006). In essence, comparative studies cannot unequivocally demonstrate causal effects of biodiversity on ecosystem functions, since apparent correlations may arise for many reasons, including the reverse relationship (e.g., ecosystem functions such as productivity altering biodiversity), or unobserved drivers affecting diversity and/or ecosystem functions. In an effort to better understand mechanisms, BEF-research has increasingly moved toward direct manipulation of diversity under otherwise constant environmental conditions, an approach that can attribute observed responses to the direct biodiversity manipulation. It is important to distinguish these two cases. Approaches based on either comparison across environmental gradients/treatments or direct manipulation of biodiversity often led to conflicting results. For example, increasing productivity caused by resource supply often leads to reduced plant diversity, mainly through enhanced competition for light, and hence apparent negative BEF relationships (Abrams, 1995; Hautier et al., 2009), a pattern that has also been reported in microbial systems (Patra et al., 2005). In contrast, diversity manipulations generally reveal positive biodiversity-productivity relationships (Balvanera et al., 2006). These seemingly contradictory results are in fact consistent when accounting for the interplays between site fertility, diversity, and productivity (Schmid, 2002).

**Table 1:** **Common terms used in BEF and trait-based BEF approaches**.

	**Definition**
Functional traits	Well-defined, measurable properties at the individual level (e.g., organisms, populations) generally used to link performance and contribution to one or several function(s) in any given ecosystem. Thereby, any key property related to physiology, morphology, or genomic information that affects the fitness or function of an organism can be regarded as a functional trait (Violle et al., 2007).
Community trait mean	Mean value calculated for each trait as the mean trait value in a community which can be weighted by the relative abundance of individual taxa in a community (Díaz et al., 2007; Violle et al., 2007)
Gradient analysis	Assessment of functioning, abundances and/or diversity of organisms along an environmental gradient in the field, or in the laboratory along pre-defined treatment gradients (McGill et al., 2006)
Ecosystem functions/functioning	Ecosystem functions in a broad sense can be categorized into functions, e.g., fluxes of energy, nutrients and organic matter; and functioning, e.g., primary production, disturbance resistance, and services like crop yield, wood production, and soil erosion control (Balvanera et al., 2006; Cardinale et al., 2012)
Application	N-dimensional hypervolume with n as the number of dimensions defining the niche, e.g., salinity, temperature, food availability (Begon et al., 2006).

Biodiversity effects on ecosystem functioning mainly arise from niche-related mechanisms that shape interactions of the biological units (e.g., OTUs, species, genotypes, ecotypes, functional groups, or phylogenetic groups) that vary genetically and in the expressed functional traits (see Table 1 for definition). These mechanisms are traditionally classified into three broad groups. First, differentiation in resource niches can lead to reduced competition, and an increased community niche size (Table 1). As a result, the associated capture of limiting resources is more efficient and community or ecosystem-level performance increases (Loreau, 2000). Such “complementarity effects” emerge from competition for resources (Salles et al., 2009), and from differences in niches related to pathogens and predation. When host-specific organisms are involved, an increase in diversity typically positively affects ecosystem function. For instance Zhu et al. (2000) demonstrated that genetically diversified rice crops had 89% greater yield, while a major rice disease had 94% less severe effects on diversified crops compared to rice monoculture controls. A second group of mechanisms is generally summarized under the term “selection effects,” represented by the probability that high diversity communities are more likely to contain species with particular traits that translate into above-average performance. Such effects are typically restricted to few species, and occur at the expense of others. Finally, “facilitation effects” occur when certain species modify environmental conditions in a way that is beneficial for other species (Bruno et al., 2003). A typical example is the presence of legumes and their nitrogen-fixing symbionts that lead to a nutrient enrichment of the ecosystem and improved performance of non-fixing plant species and nitrogen-related microbial processes (Spehn et al., 2005; Le Roux et al., 2013).

BEF relationships ultimately arise from functional differences among the biological units of which communities are comprised. For instance, in plant communities, functional diversity was the driving factor explaining plant productivity (Tilman, 1997). In another study Norberg et al. (2001) introduced a framework that suggests a linear relationship between variances in phenotypes within functional groups and responses to environmental changes. A later example focused on the role of functional diversity to explain BEF relationships and whether or not this is linked to phylogenetic diversity (Flynn et al., 2011). However, functional traits and the resulting ecological niches are the determinants of species interactions and consequently ecosystem functioning. Traits refer to the physiology, morphology, or genomic characteristics that affect the fitness or function of an organism. Traits can be used to infer its performance under different environmental conditions (Violle et al., 2007), they can be measured or scaled-up at the community level, and eventually be related to community and ecosystem functioning (Violle et al., 2007; Wallenstein and Hall, 2012).

**Figure 1:**
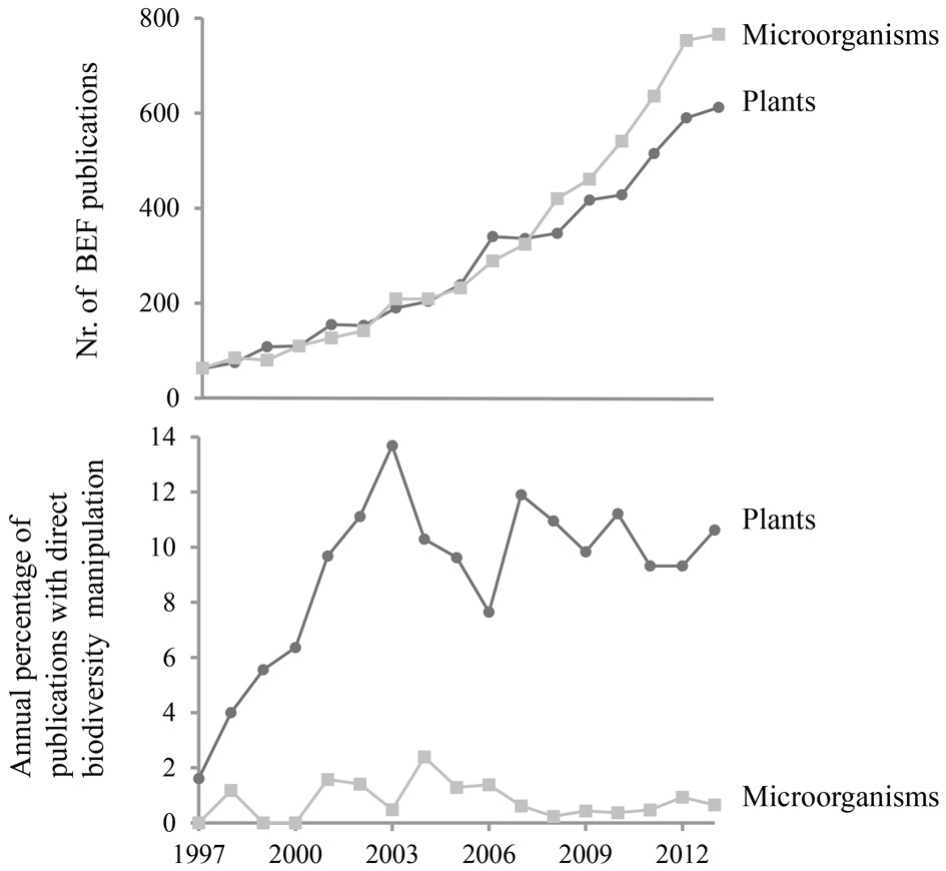
**Temporal variations in (top) the number of publications on Biodiversity-Ecosystem Functioning, BEF, relationships in a broad sense for microorganisms as compared to plants, and (bottom) the percentage of publications on microbial BEF or plant BEF where biodiversity was directly manipulated**. The search terms used are provided in Supplementary Material 1. At each step of the search profile development, we checked on subsamples that the search hits corresponded to the targeted type of studies. We also checked that a selection of key experiments/papers we knew about were found.

Meta-analyses clearly demonstrated that the relationship between biodiversity and ecosystem functioning has primarily been studied for higher organisms (Balvanera et al., 2006; Cardinale et al., 2012). A systematic search of published papers which refer to microbial diversity and ecosystem functioning nevertheless shows that the total number of papers is quite similar for plant- and microbe-related studies identifying the analysis of BEF relationships as a key objective (Figure 1). However, a closer examination reveals that most microbial BEF studies rely on comparative designs where biodiversity is not directly manipulated (Figure 1).

Microbial BEF research is evolving rapidly (Allison and Gessner, 2012; Bouskill et al., 2012) but microbial ecologists often quantify traits at scales ranging from populations (e.g., physiological characteristics of strains) to communities (e.g., functional gene pools or substrate utilization patterns from environmental samples) and rarely consider existing trait-related concepts to evaluate BEF relationships as used in ecology. Trait-based approaches could be particularly useful in microbial ecology by complementing microbial approaches based on taxonomy or functional gene/protein sequence diversity and enhancing our ability to link microbial diversity to the functioning of microbial communities and ecosystems.

We review microbial studies relating diversity and process rates, focusing more particularly on the application of trait-based approaches, and identify their current progress and pitfalls. We distinguish the application of trait-based approaches for comparative studies across environmental gradients/treatments (Table 1), and BEF-studies in which biodiversity is manipulated directly (Table 2). We highlight why trait-based approaches could spur significant progress in the understanding of microbial BEF relationships in the future and evaluate how traits can be more directly incorporated into microbial BEF studies. Finally, we discuss the potential and challenges of microbial trait-based approaches to promote the emergence of principles in microbial ecology and BEF relationships in general.

**Table 2:** **Comparison between trait-based studies that relate microbial biodiversity and ecosystem functioning across environmental gradients/treatments, and those directly manipulating components of diversity**.

	**Comparative studies**	**Manipulated diversity studies**
Level of trait assessment	Functional group/Community	Strain
Trait resolution	Community-mean traits/within community distribution of traits	Taxon-specific traits/multiple traits in individual taxa/tradeoffs among traits
Key eco-physiological techniques	Stable isotope probing; Biolog/Ecoplates; etc.	Metabolic and physiological studies of individual cells and strains
Key -omics techniques	DNA and RNA single gene sequence diversity; environmental (meta-)genomics, transcriptomics, proteomics, and metabolomics	Genomics, transcriptomics, proteomics, and metabolomics on cells and strains
Main scale	The real world (field studies; complex natural communities)	Laboratory (model systems)
Level of understanding	Correlational link between biodiversity and functioning along environmental gradients	Causal/direct/mechanistic link between biodiversity and functioning; complementarity/selection/facilitation effects

## Distinction of BEF relationships in microbial systems

### Comparative investigation of microbial BEF relationships

There are many examples where bacterial composition changes along environmental gradients (Hughes Martiny et al., 2006; Fierer et al., 2007; Van Der Gucht et al., 2007; Attard et al., 2010; Nemergut et al., 2011; Newton et al., 2011; Ghiglione et al., 2012). However, it is often difficult to mechanistically understand the observed correlation between diversity and function in such comparative approaches, because diversity is an observed, dependent variable rather than an applied treatment. Moreover, many environmental parameters can co-vary with diversity, driving observed relationships.

It is particularly difficult to explain such correlations between microbial diversity and ecosystem function in relation to functional diversity. Many bacterial groups are not available in pure culture, which hinders determination of their physiology and consequently assessment of their functional roles in aquatic and terrestrial environments. Recent evidence suggests that a potentially large portion of the microbial diversity detected in gradient studies are not directly contributing to function, being either dead, in a dormant state or present as extracellular DNA (Lennon and Jones, 2011; Blagodatskaya and Kuzyakov, 2013). Although the “dormant diversity” is part of a microbial seed bank from which different traits can be resuscitated (Lennon and Jones, 2011), it can obscure environmental microbial BEF studies. The use of isotope probing (SIP) represents a way to single out taxa that are actively contributing to function while accounting for non-active, members of a community (Bodelier et al., 2013).

It is crucial to relate a particular process to the diversity of the respective, functionally coherent group; such an analysis has the potential for successfully detecting causal links between microbial diversity and ecosystem function. For instance, some studies reported clear relationships between the diversity of soil ammonia- (Webster et al., 2005) or nitrite-oxidizers and nitrification across management practices in relation to the availability of inorganic nitrogen (Attard et al., 2010). The abundance of soil *Nitrobacter*, which are nitrite-oxidizing bacteria with high growth rate/specific activity and low N substrate affinity, increased along a nitrogen gradient (Attard et al., 2010). In contrast, the abundance of *Nitrospira*, which are nitrite-oxidizing bacteria with low growth rate/specific activity and high N substrate affinity tended to decrease along this gradient. While in this case both changes in diversity and functioning of nitrite-oxidizers respond to changes along an environmental gradient, diversity changes are important in allowing function to increase with increased N availability. Using a number of traits derived from eco-physiological studies with various guilds of nitrifiers, a trait-based modeling framework successfully predicted a number of functions (i.e. ammonia oxidation, N_2_O emission) in published datasets in various environmental gradients (Bouskill et al., 2012). Of course, this study heavily relies on the coverage of nitrifier diversity by cultured representatives and associated trait information.

A trait-based perspective can facilitate the handling and interpretation of microbial diversity along environmental gradients by measuring functional traits under the specific conditions a given community is exposed to, i.e. as “realized community mean traits.” This differs from “a priori” trait values of organisms measured under standardized conditions, and will in part mirror responses to the specific environment and the specific diversity of the community.

### Direct manipulation of diversity to study microbial BEF relationships

Microbial BEF relationships can also be studied by analyzing the effect of a targeted reduction in microbial biodiversity, e.g., in soil and aquatic microcosms (Le Roux et al., 2011). For instance, reductions in the diversity of pasture soil communities by progressive fumigation or serial dilution had no consistent effect on a range of soil processes (Griffiths et al., 2000, 2004). The removal of diversity for key microbial functional groups such as nitrifiers or denitrifiers provided important information on the extent of functional redundancy within these functional groups (Wertz et al., 2007; Philippot et al., 2013). Reduction of diversity in aquatic microbial communities clearly showed that some metabolic functions (i.e., chitin and cellulose degradation) were controlled by single phylotypes and their traits rather than by richness of the total community (Peter et al., 2011), whereas other functions such as growth were positively correlated to richness. It has to be noted that removal experiments prescribe particular scenarios of diversity loss (e.g., a suspension/dilution approach implies that less abundant species are removed first) which are important for effects on ecosystem functioning (Jones and Lennon, 2010).

An additional step toward understanding the functional role of microbial diversity stems from studies assembling communities through the combination of microbial populations, for example by random selection from a source species pool. This so-called “assemblage approach” has already been used to describe how the diversity of fungal communities influence litter decomposition (Janzen et al., 1995; Cox et al., 2001), the role of mycorrhizal fungal diversity on plant productivity (Van Der Heijden et al., 1998; Jonsson et al., 2001), the role of bacterial diversity on cellulose degradation (Wohl et al., 2004), the role of evenness on the stability of microbial ecosystem functions (Wittebolle et al., 2009), and the role of soil bacterial diversity on mineralization or denitrification (Bell et al., 2005; Salles et al., 2009).

Assemblage experiments offer opportunities to identify mechanisms that may underlie microbial BEF relationships (Le Roux et al., 2011). In particular, functional traits of the assembled strains can be characterized, providing information on whether trait complementarity or selection are major mechanisms for explaining observed BEF relationships (Roscher et al., 2012). For instance, key traits among denitrifying bacteria were linked to the use of different carbon (C) sources that strongly determined the functioning of assembled communities on a mix of C sources (Salles et al., 2009, 2012). The complementarity for traits was a much better predictor of denitrification than taxa richness, the phylogenetic diversity of the communities based on *16S rRNA* gene sequences, or even the diversity assessed by functional gene/protein sequences (Salles et al., 2012). In contrast, antagonistic controlling mechanisms were observed for assembled communities of *Pseudomonas fluorescens*, where inhibition of strains determined the performance of the assembled community (Jousset et al., 2011).

One shortcoming of assemblage experiments is that the assembled, e.g., bacterial communities rarely exceed 100 taxa and hence the diversity is very low compared to the richness observed in most natural communities. Besides, only culturable microorganisms can be used to assemble these communities, even though culture-independent studies suggest the importance of taxa in ecosystem functioning that have not been cultivated (Chen et al., 2008; Mackelprang et al., 2011; Iverson et al., 2012). Nevertheless, studies employing direct manipulation of biodiversity by removal or random assembly of microbial populations remain scarce and represent less than 1% of published microbial studies focusing on the relationship between diversity and ecosystem functioning (Figure 1). We believe that an increased effort to couple trait-based approaches and assemblage experiments could be a very powerful strategy to specifically identify and decipher the mechanisms underlying microbial BEF relationships.

## Trait-based approaches to advance microbial BEF studies

### Integrating trait-based and phylogenetic/taxonomic approaches to understand microbial BEF

Prior to development and adoption of phylogenetic based tools, bacterial taxonomy was based on phenotypes and physiological characteristics that could only be measured in pure cultures (Staley, 2006). Today, the availability of large databases of marker genes (e.g., the Ribosomal Database Project or Greengenes) has enabled the establishment of a detailed classification scheme for microorganisms that also includes those groups that we have not yet been able to cultivate. However, for studying microbial BEF relationships, a classical taxonomic/phylogenetic approach is hampered by the current species definition (Schleifer, 2009) which can demarcate taxonomic units—which can still be enormously diverse both in functionality and ecology (Staley, 2006; Green et al., 2008). In our opinion, the inherent limitations with regards to the concept of microbial species are not the major issues here, and two other factors are of much more central importance.

To understand BEF relationships it is necessary to study traits at the level of individual cells or organisms (Lavorel et al., 2013). The niches that correspond to traits are hyper-dimensional, and BEF studies call for determining whether niches of functional units overlap. To fully appreciate functional diversity, whether assessed as richness, divergence or dispersion of traits (Hedberg et al., 2013), one has to characterize and account for trade-offs among the different traits. For example, plant leaf trait trade-offs have been shown to affect litter decomposition and therewith the incidence of wild fires (Brovkin et al., 2012), whereas trade-offs for key traits among bacterial decomposers can restrict the bacterial degradation of recalcitrant carbon to sites with high nitrogen availability (Treseder et al., 2011) and influence how bacteria contend with other abiotic factors such as moisture variability (Lennon et al., 2012). However, characterizing functional trait values and quantifying trade-offs only may lead to spurious correlations and there are only a few examples that demonstrate actual trade-offs supported by plausible physical or chemical mechanisms (e.g., Edwards et al., 2011). Hence, knowledge about trade-offs is indispensable for accurate descriptions of functional BEF relationships and necessitates identification of relevant functional units such as species, ecotypes, or genotypes.

**Figure 2:**
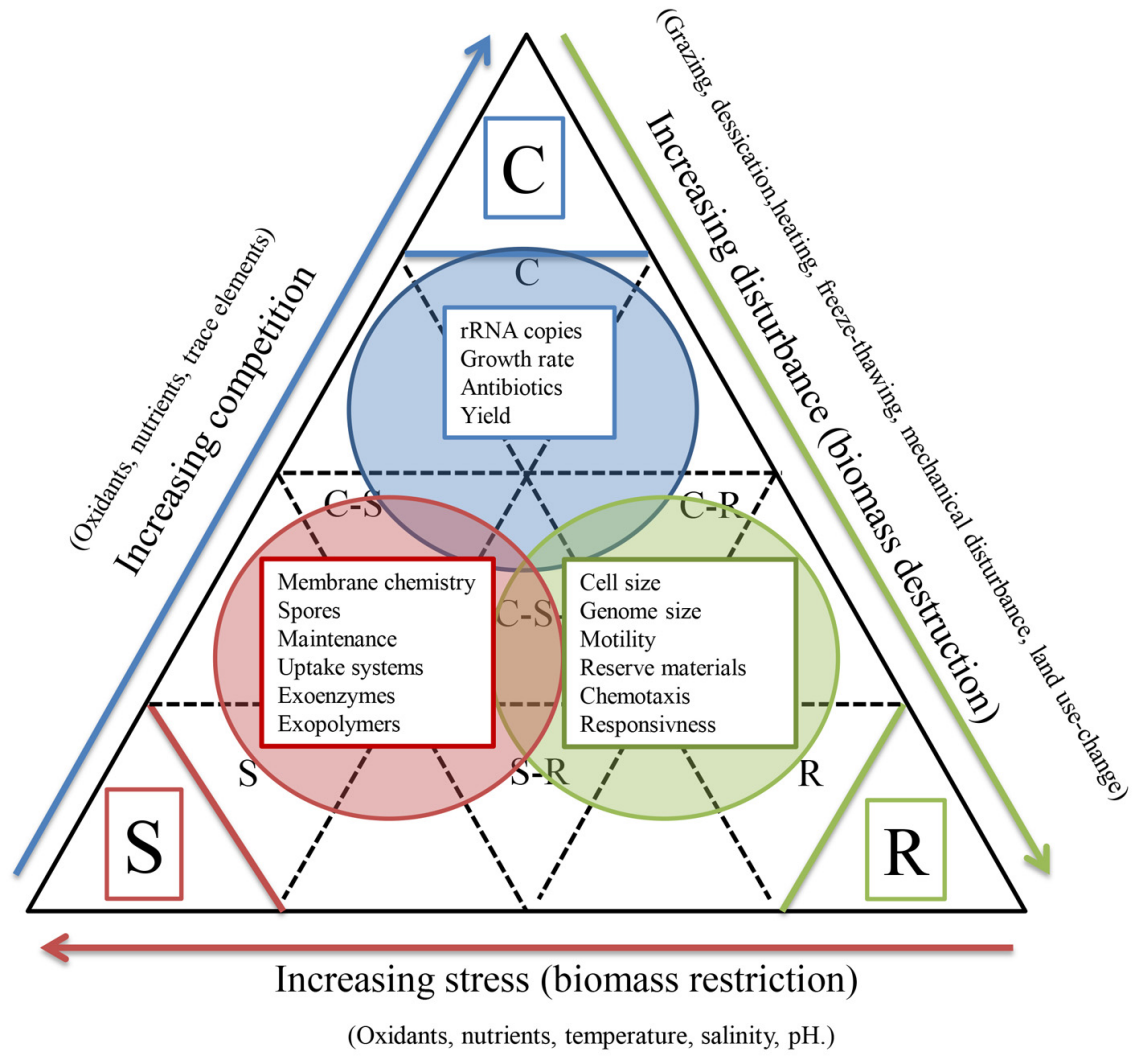
**Reflection of microbial traits on the Competitor-Ruderal-Stress tolerator life strategy framework as was proposed for plants (Grime, 1977)**. The scheme has been adapted for Ho et al. (2013) who used this framework for assigning life-strategies to methane-oxidizing bacteria. The scheme groups subsets of microbial traits which collectively would be of most importance for the respective strategy. The traits collectively accommodate exploring and exploiting habitats, competing with other organisms, tolerating or avoiding surviving stress, and deprivation. This classification is purely qualitative but, for some traits, life-history strategies have been proposed in earlier studies (Fierer et al., 2007; Portillo et al., 2013).

The relevance of trade-offs among microbial traits is recognized (Litchman et al., 2007), but better characterizing trade-offs among microbial traits are likely to be of increasing importance for microbial BEF studies for several reasons. First, they aid in reducing the number of functional dimensions that need to be considered. Second, the co-occurrence of traits and trade-offs help to define microbial strategies beyond the familiar *r* vs. *K* strategies. For example, the life-history scheme designed for plants (Grime, 1977) was used to classify methane-oxidizing bacteria according their competitive ability, ruderal and stress tolerating properties based on culture and environmental traits (Ho et al., 2013). This conceptual approach combines information about phylogeny and function and aggregates traits into community responses, allowing for mixed life strategies and offering more flexibility to accommodate the vast metabolic flexibility of bacteria (Figure 2). Though, extrapolation of this conceptual framework to microbial communities deserves experimental validation. There is considerable debate regarding the coherence between phylogeny and the distribution of functional traits (Losos, 2008). If traits are conserved to some degree throughout evolution (trait conservatism), phylogenetic diversity could be a promising proxy for assessing trait diversity. For instance, Cadotte et al. (2008) analyzed 29 studies in which angiosperm biodiversity was manipulated in a systematic way and found that phylogenetic diversity indices explained significantly more variation in productivity than plant species richness or other diversity measures that were available. Flynn et al. (2011) analyzed data from 29 experiments involving 174 plant species that were present in 1721 combinations and found that functional trait diversity and phylogenetic diversity explained similar amounts of variation in the observed responses. Interestingly, phylogenetic diversity explained variation in data that was not explained by traits, suggesting that it is a surrogate to quantify trait differences along niche axis that are difficult to assess directly (such as pathogen-related niches, or complex hyper-dimensional combinations of single traits assessed).

In microbial ecology, the extent to which functional traits are phylogenetically conserved remains unclear. Considering the rather extensive horizontal gene transfer (Polz et al., 2013) likely compromising a unifying phylogenetic framework, functional diversity measures do not necessarily follow either taxonomy, phylogenetic or apparent evolutionary relationships. For instance, variations in key functional traits of denitrifying bacteria were not well correlated to their (*16S rRNA*-based) phylogenetic relatedness or functional gene/protein sequence relatedness (Jones et al., 2011; Salles et al., 2012). From another perspective, several studies reported broad ecological coherence of high bacterial taxonomic ranks building on *16S rRNA* phylogeny (Fierer et al., 2007; Von Mering et al., 2007; Philippot et al., 2009; Lennon et al., 2012). The question thus remains whether or not one should abandon taxonomy- and phylogeny-based approaches altogether for studies of BEF relationships. If traits are phylogenetically conserved at least for some microbial groups, phylogenetic diversity could serve as proxy for functional diversity. Calculating functional diversity indices generally requires the assessment of traits at the individual or some aggregated taxonomic level, which generally is impossible in microbial studies that do not build assemblage-based designs. Martiny et al. (2012) recently developed a new phylogenetic metric which estimates the clade depth of shared traits between organisms. This approach could be used to translate differences in community composition into consequences for microbial-mediated processes. Another approach models evolutionary dynamics of bacteria to ecologically distinct lineages, so called ecotypes, within natural communities, allowing for a highly resolved ecological classification (Koeppel et al., 2008). Such distinction of microbial taxa based on ecological features would bridge the gap between taxonomy- and trait-based approaches in microbial ecology. We argue that trait-based approaches should build on—not replace—taxonomy-based approaches. The information needed to properly characterize the co-occurrence of traits and trait trade-offs among microorganisms builds on taxonomic ranks, and there is certainly an incentive for more high-throughput surveys of phenotypic characteristics of microbial taxa (Bayjanov et al., 2012). Such approaches could mark the beginning of a deviation from classical phylum-based approaches in microbial BEF studies toward a classification based on functional performance and role in the environment.

### Tools available to integrate traits into microbial BEF studies

Measurements of taxonomic microbial diversity are very challenging since diversity levels are extremely high for most natural microbial systems (Torsvik et al., 2002; Caporaso et al., 2011). To obtain functional diversity measures in microbial BEF studies, the biggest challenges are (i) defining which microbial traits are important with respect to ecosystem functioning or particular ecosystem functions, and (ii) measuring these relevant traits.

For the assemblage studies, microbial ecologists can measure multiple traits for individual microorganisms or populations and quantify tradeoffs between traits. However, defining the types of relevant traits to measure is a challenge, depending on the community functioning under study. On the other hand, traits can be related to shifts in function across environmental gradients or treatments, at the genetic or functional level, or directly at the community scale. However, this is different from analyzing BEF-relationships in the general ecological context, which requires methods capable of quantifying the local functional diversity (i.e., the variation of trait combinations present at the individual level). Community mean traits are not useful for this purpose, since the information about effects of the local trait diversity (i.e., the putative local driver of a BEF-relationship) will be lost by averaging.

The analysis of metabolic processes offers great potential to evaluate aggregated trait values at the community scale. Functional traits can be assessed by high-throughput assays, such as Biolog or Ecoplates. These cultivation-based metabolic assays can be used to characterize the community capacity to oxidize a range of C sources (Garland, 1996) or to measure a functional operating range of soil or aquatic microbial communities (Hallin et al., 2012).

We see some advantage for microbial studies correlating community-mean traits to functional capabilities of the community as a whole, since these are more easily measurable than for higher organisms. Indeed, aggregated trait values would boil down to a metric sizing of “meta-species,” which can illuminate responses along environmental gradients and possible effects on ecosystem functioning. A drawback is that the combination of traits of all microbial individuals that compose the community can hardly be characterized.

We can expect that our ability to identify and quantify functional traits of microbial individuals and populations in natural, complex communities will increase in the coming years. Despite the dogma that we cannot study the physiology of ecologically “relevant” microbes from environmental samples owing to the challenges associated with the enrichment and isolation of most taxa, we must recognize that there have already been major advances in cultivation efforts over the past 20 years. *In situ* enrichments (e.g., diffusion chambers and baited beads) and other incubation methods can be used to determine cell-specific metabolic rates, even at extremely low rates (Hoehler and Jorgensen, 2013). Additional physiological features that are tractable today (without isolation) include cell-size related nutrient affinity and nutrient use efficiency (Edwards et al., 2012), and specific substrate use with isotope tracking methods targeting single cells of different size and shape (flow cytometry and stable isotope tracers, microautoradiography, nano-SIMS) (Nielsen et al., 2003; Casey et al., 2007; Behrens et al., 2012; Garcia et al., 2013). In addition, just like in the omics realm, there have been major advances in microscopy and bio-molecular imaging over the last 20 years (Haagensen et al., 2011), with novel and refined techniques that offer huge opportunities to access key aspects of functional diversity, even within complex microbial communities. For instance, we can now have access to the bulk biochemical composition of cells by RAMAN spectroscopy (Huang et al., 2007), and to their spatial organization (Stiehl-Braun et al., 2011). Sensitive fluorescence-based techniques enable visualization of novel morphological and physiological features (porins, flagella, proteins, and protein-coding genes etc.) and of associations based on syntrophic interactions (Watrous et al., 2013). Hence, even if a proper quantification of the functional diversity of natural, complex communities from multiple trait values of individuals composing these communities remains challenging, a toolkit already exists to help microbial ecologists working in this domain. Finally, the dramatically improved opportunities to reconstruct genomes of so far uncultivated microbial populations and cells by binning of complex metagenomes (Rusch et al., 2010; Iverson et al., 2012) has demonstrated great potential for resolving metabolic and functional traits of uncultured and poorly known representatives in the microbial world (Wrighton et al., 2012). This can even be combined with *in situ* substrate usage of uncultivated microbes (Mayali et al., 2012). Single cell genome sequencing (Stepanauskas, 2012) is another feasible way to elucidate and infer genome encoded traits in uncultured microbial populations that often make up the bulk portion of natural communities and are likely to have a large impact on ecosystem functions.

We believe that microbial ecologist have the ability to provide new insights to trait-based ecology as opposed to just borrowing ideas and approaches from other non-microbial ecologists, fully making use of the particularities of microbial systems and tools. In particular, microbial ecology should play a key role in deciphering the effects of functional diversity and spatial distribution in BEF studies, offering very relevant and manageable models to address this key issue.

## Conclusion and perspectives

Microbial communities are a key variable in how natural and anthropogenic disturbances, including climate change, will affect ecosystem functioning and hence delivery of services to human societies. The trait-based approach is not the Holy Grail (Lavorel and Garnier, 2002) but a promising framework and discourse for future microbial research. In particular, promising experimental approaches that incorporate functional traits can pave the road to increase the understanding of microbial BEF relationships, and BEF relationships in general.

Microbial ecologists face challenges but also great opportunities in this context. Instead of simply suggesting the need to renew approaches in BEF research using traits, we argue that two main priorities for microbial BEF studies are (i) to reinforce experimentally-sound studies of the role of microbial (trait-based) diversity on ecosystem functioning, and (ii) to promote efforts for measuring and archiving microbial traits in a way suitable for the highly diverse and dynamic microbial communities that make up the biosphere.

The first priority arises from the current paucity of microbial ecology in terms of BEF studies that directly manipulate diversity using a trait-based approach. While assembled communities clearly differ from complex communities from natural environments, this does not diminish the value and potential of such studies to disentangle the possible key mechanisms underlying BEF relationships.

Concerning the second priority, we call for more innovative physiological studies in order to measure traits and their relevant unit (e.g., single strains, population, or community-level). More specifically, by measuring traits in a standardized manner, e.g., incubation condition and media, and by applying analogous tests also to organisms we cannot get in pure culture, we may be able to reveal important trait distributions and generate a microbial trait database similar to, e.g., the TRY global traits initiative for plants (Kattge et al., 2011). Microbial ecologists can also capitalize on novel powerful genome sequencing tools being applied to communities or single uncultured cells, which may serve as a tool for predicting ecosystem function from detected (genomic) traits (Raes et al., 2011; Barberan et al., 2012).

Microbial ecologists can provide new insights and concepts to trait-based BEF studies, according to the particularities of microbial systems and the tools available in microbial ecology. For instance, BEF studies over many microbial generations allow researchers to reveal the effect of eco-evolutionary feedbacks on BEF relationships over reasonable time scales. Also, accounting for spatial and temporal niche variability as well as assessing the role of diversity in multiple related ecosystem functions, microbial trait-based approaches may deliver mechanistic insights in areas practically not feasible in higher organisms, thus providing benefits to ecology as a whole, which is still a major challenge for microbial ecologists (Prosser et al., 2007).

### Conflict of interest statement

The authors declare that the research was conducted in the absence of any commercial or financial relationships that could be construed as a potential conflict of interest.
