# Supplementary material for: Trait-based approaches for understanding microbial biodiversity and ecosystem functioning
Source: Front Microbiol. 2014 May 27;5:251. doi: 10.3389/fmicb.2014.00251 (PMC4033906; doi:10.3389/fmicb.2014.00251)
Supplement: Supplementary file 1 [file DataSheet1.PDF]

Supplementary Material

Supplemental material to Figure 1: Data collection.

<u>(1) To characterize the temporal variations in the number of publications on Biodiversity-Ecosystem Functioning, BEF, relationships in a broad sense for plants, the Web of Science database was searched for the following profile until 31 December 2013:</u>

((“plant diversity”) or (”plant biodiversity”) or (“plant richness”) or (“plant species richness”) or (“plant species“ and “diversity”) or (“plant functional diversity”) or (”plant functional composition ”) or (“plant genotype*“) or (“diverse plant communities“) or (“diverse grassland communities“) (“plant assemblages“) or (“plant community diversity“) or (“diversity of grassland communities“) or (“species richness of plants“) or (“tree genotype*“) or (“tree diversity”) or (“tree richness”) or (“tree species richness”) or (“tree species diversity”) or (“tree functional diversity”) or (“grassland species“ and “species richness”) or (“grassland species“ and “species diversity”) or (“forest species“ and “species richness”) or (“forest species“ and “species diversity”) or ((“functional traits“ or “functional trait diversity“ or “functional groups“) and (plant or tree or grassland or forest))) and (“ecosystem functioning“ or “ecosystem functions“ or “ecosystem processes“ or “community functioning“ or “community functions“ or “community processes“ or productivity or invasibility or colonizer* or invader* or (“functioning of ecosystems“) or (“ecosystem properties and processes“) or (“resource use“) or (“nutrient acquisition“) or (“light acquisition“) or (“biomass“) or (“primary production“) or ((yield or yields) and crop*) or (“ecosystem effects of biodiversity“) or (“role of biodiversity in ecosystems“))

<u>(2) To characterize the temporal variations in the number of publications on BEF relationships in a broad sense for microorganisms, the Web of Science database was searched for the following profile until 31 December 2013:</u>

((“bacteria* diversity”) or (“bacteria* biodiversity”) or (“bacteria* richness”) or (“bacteria* species richness”) or (“bacteria* species diversity”) or (“bacteria* species“ and “diversity”) or (“bacteria* functional diversity”) or (”bacteria* functional composition ”) or (“bacteria* genotype*“) or (“bacteria* genetic diversity“) or (“diverse bacteria* communities“) or (“bacteria* assemblages“) or (“bacteria* community diversity“) or (“diversity of bacteria* communities“) or (“species richness of bacteria*“) or (“fung* diversity”) or (“fung* biodiversity”) or (“fung* richness”) or (“fung* species richness”) or (“fung* species diversity”) or (“fung* species“ and “diversity”) or (“fung* functional diversity”) or (”fung*

functional composition ”) or (“fung* genotype*“) or (“fung* genetic diversity“) or (“diverse fung* communities“) or (“fung* assemblages“) or (“fung* community diversity“) or (“diversity of fung* communities“) or (“species richness of fung*“) or (“archaea* diversity”) or (“archaea* biodiversity”) or (“archaea* richness”) or (“archaea* species richness”) or (“archaea* species diversity”) or (“archaea* species“ and “diversity”) or (“archaea* functional diversity”) or (”archaea* functional composition ”) or (“archaea* genotype*“) or (“archaea* genetic diversity“) or (“diverse archaea* communities“) or (“archaea* assemblages“) or (“archaea* community diversity“) or (“diversity of archaea* communities“) or (“species richness of archaea*“) or (“microbial diversity”) or (“microbial biodiversity”) or (“microbial richness”) or (“microbial species richness”) or (“microbial tax* richness”) or (“microbial species diversity”) or (“microbial tax* diversity”) or (“microbial species“ and “diversity”) or (“microbial tax*“ and “diversity”) or (“microbial functional diversity”) or (”microbial functional composition ”) or (“microbial genotype*“) or (“microbial genetic diversity“) or (“diverse microbial communities“) or (“microbial assemblages“) or (“microbial community diversity“) or (“diversity of microbial communities“) or (“species richness of microorganisms“) or (“tax* richness of microorganisms“) or (“strain diversity“) or (“diversity of strains“) or ((biodiversity) and (bacteria* or fung* or archaea*)) or ((diversity) and (bacteria* or fung* or archaea*))) and (“ecosystem functioning“ or “ecosystem function“ or “ecosystem functions“ or “ecosystem processes“ or “community functioning“ or “community functions“ or “community processes“ or productivity or invasibility or colonizer* or invader* or (“functioning of ecosystems“) or (“ecosystem properties and processes“) or (“resource use“) or (“soil fertility“) or (“primary production“) or ((yield or yields) and crop*) or (“ecosystem effects of biodiversity“) or (“role of biodiversity in ecosystems“) or nitrification or denitrification or (“nitrogen fixation“) or (“methane oxidation“) or (“methane production“) or (“soil respiration“) or (“plant growth“) or (“soil functions“) or (“soil biological functions“) or (“soil functions“) or biofiltration or (“resistan* to stress*“) or (“resistan* to disturbance*“) or (“resistan* to perturbation*“) or (“resilience capacity“))

<u>(3) To depict the percentage of publications on plant BEF where biodiversity was directly manipulated, the search corresponding to profile (1) was further restricted using the following profile search :</u>

(“assemblage experiment” or (“assembled communit*”) or (“assembled plant communit*”) or (“assembled grassland communit*”) or (“removal experiment”) or dilution or (“diversity manipulation”) or (”diversity experiment”) or (monoculture* and mixture*) or (“factorial species richness“) or (“biodiversity experiment“) or (“biodiversity experiments“) or (“sown plant diversity“) or (“field experimental plots“) or (“experimental plant communities“) or (“experimental communities“) or (“experimental grassland*“) or (“experimentally established plant communities“) or (“experimental grassland systems“) or (“manipulating plant diversity“) or (“species assembled“) or (“experimental plant assemblages“) or (“primary producer manipulations“) or (“biodiversity manipulations“) or (“constructed grassland

ecosystems“) or (“synthesizing grassland communities“) or (“experimental diversity gradients“))

(4) To depict the percentage of publications on microbial BEF where biodiversity was directly manipulated, the search corresponding to profile (2) was further restricted using the following profile search (key papers were also identified by co-authors):

(“assemblage experiment” or (“assembled communit*”) or (“assembled bacteria* communit*”) or (“assembled fung* communit*”) or (“assembled microbial* communit*”) or (“removal experiment”) or (“dilution approach”) or (“removal approach”) or (“dilution of a soil suspension”) or (“serially diluted soil suspensions”) or (“diversity manipulation”) or (”diversity experiment”) or (“factorial species richness“) or (“inoculated bacteria* diversity“) or (“inoculated fung* diversity“) or (“inoculated microbial diversity“) or (“experimental bacteria* communities“) or (“experimental fung* communities“) or (“experimental microbial communities“) or (“experimental communities“) or (“experimental microbial systems“) or (“experimental bacteria* systems“) or (“experimental fung* systems“) or (“manipulating bacteria* diversity“) or (“manipulating fung* diversity“) or (“manipulating microbial diversity“) or (“species assembled“) or (“strains assembled“) or (“experimental bacteria* assemblages“) or (“experimental fung* assemblages“) or (“experimental microbial assemblages“)or (“biodiversity manipulations“) or (“synthesizing bacteria* communities“) or (“synthesizing fung* communities“) or (“synthesizing microbial communities“) or (“experimental diversity gradient*“) or (microbial diversity was reduced using serial dilutions) or (dilution of a soil suspension prepared from unsterilized soil) or (microcosms of varying lineage and phylogenetic diversity) or (experimental communities of five bacterial species) or (Assemblages of generalists) or (manipulate large numbers of bacterial species selected at random)
